# Practices, patients and (im)perfect data - feasibility of a randomised controlled clinical drug trial in German general practices

**DOI:** 10.1186/1745-6215-12-91

**Published:** 2011-04-01

**Authors:** Ildikó Gágyor, Jutta Bleidorn, Karl Wegscheider, Eva Hummers-Pradier, Michael M Kochen

**Affiliations:** 1 Department of General Practice and Family Medicine, University of Goettingen, Humboldtallee 38, 37073 Goettingen, Germany; 2 Institute of General Practice, Hannover Medical School, Carl-Neuberg-Str.1, 30625 Hannover, Germany; 3 Department of Medical Biometry and Epidemiology, University Medical Centre Hamburg-Eppendorf, Martinistr. 52, 20246 Hamburg, Germany

## Abstract

**Background:**

Randomised controlled clinical (drug) trials supply high quality evidence for therapeutic strategies in primary care. Until now, experience with drug trials in German general practice has been sparse. In 2007/2008, the authors conducted an investigator-initiated, non-commercial, double-blind, randomised controlled pilot trial (HWI-01) to assess the clinical equivalence of ibuprofen and ciprofloxacin in the treatment of uncomplicated urinary tract infection (UTI). Here, we report the feasibility of this trial in German general practices and the implementation of Good Clinical Practice (GCP) standards as defined by the International Conference on Harmonisation (ICH) in mainly inexperienced general practices.

**Methods:**

This report is based on the experience of the HWI-01 study conducted in 29 German general practices. Feasibility was defined by 1) successful practice recruitment, 2) sufficient patient recruitment, 3) complete and accurate data collection and 4) appropriate protection of patient safety.

**Results:**

The final practice recruitment rate was 18%. In these practices, 79 of 195 screened UTI patients were enrolled. Recruitment differed strongly between practices (range 0-12, mean 2.8 patients per practice) and was below the recruitment goal of approximately 100 patients. As anticipated, practice nurses became the key figures in the screening und recruitment of patients. Clinical trial demands, in particular for completing symptom questionnaires, documentation of source data and reporting of adverse events, did not agree well with GPs' documentation habits and required support from study nurses. In many cases, GPs and practice staff seemed to be overwhelmed by the amount of information and regulations. No sudden unexpected serious adverse reactions (SUSARs) were observed during the trial.

**Conclusions:**

To enable drug trials in general practice, it is necessary to adapt the setup of clinical research infrastructure to the needs of GPs and their practice staff. Risk adaption of clinical trial regulations is necessary to facilitate non-commercial comparative effectiveness trials in primary health care.

**Trial Registration:**

Trial registration number: ISRCTN00470468

## Background

Randomised controlled clinical (drug) trials (RCTs) provide high-quality evidence for therapeutic decisions in all fields of medicine. For primary care research, drug RCTs, especially without commercial interests, are of particular relevance for two reasons:

First, primary care health conditions and their treatment should be investigated in their specific setting, since evidence from specialised structures with selected patients cannot necessarily be transferred without being re-evaluated. Second, compared with drug studies in specialised settings, research questions in primary care usually address a different issue (e.g. "step-down-therapies", less invasive and expensive therapies, or comparative effectiveness of established treatments) and focus on general populations with low disease prevalence, and a symptom-oriented approach.

Non-commercial drug RCTs have increasingly been performed in several European countries, especially in UK and The Netherlands [1-4]. Recently, several randomised controlled educational intervention studies have been conducted in German general practice [5-7], but drug trials remain scarce.

In 2007/2008, we conducted a non-commercial, double-blind, randomised controlled trial (HWI-01), which aimed to test the clinical equivalence of a three-day treatment course of 3 × 400 mg ibuprofen compared to 2 × 250 mg ciprofloxacin for uncomplicated urinary tract infection [8]. Following a mandatory requirement of the funding organisation (German Federal Ministry of Education and Research) to prove feasibility of the study design, the investigation was conducted as a pilot trial with limited sample size. Further information about the trial is given in table 1.

**Table 1:** Antibiotics vs. ibuprofen for the treatment of uncomplicated urinary tract infection:A clinical trial in general practices

Protocol number	HWI-01
**EudraCT registration**	2006-006398-26

**Trial sites**	• 19 general practices in and around Hannover
	• 12 general practices in and around Göttingen

**Time period**	2007/2008

**Trial design**	• Double blind, multicentre, randomised controlled clinical equivalence trial, investigator initiated

**Objectives**	• To describe a first trend concerning the equivalence of ibuprofen and ciprofloxacin in the treatment of uncomplicated urinary tract infection
	• To optimize documents and procedures of a double-blind, randomised-controlled trial in German general practices
	• To assess the number of treatment failures within the ibuprofen group

**Condition**	• Acute uncomplicated urinary tract infection

**Endpoints**	• Symptom resolution on day 4/7, symptom relief on day 4
	• Treatment failure in the ibuprofen group

**Number of patients**	• 79 patients were included

**Inclusion criteria**	• Women aged ≥ 18 years, written informed consent
	• Symptoms of urinary tract infection (dysuria, frequency, urgency, possible low abdominal pain)

**Exclusion criteria**	• Any signs indicating a complicated UTI (i.e. fever, back pain)
	• Any conditions that may lead to complicated infections (i.e. pregnancy, diabetes, renal diseases, urinary tract abnormalities or past urinary surgery, urine catheterization, immunosuppressive therapy, other serious diseases, cancer),
	• Previous urinary tract infection within the last two weeks,
	• Current use of antibiotics or non-steroidal anti-inflammatory drugs,
	• History of gastrointestinal ulcers; epilepsy, allergies or other
	• contraindications for trial drugs,
	• Inability to understand the trial information or to give informed consent

**Ethics approval**	• Research Ethics Committee of the University of Goettingen Medical Center (2007/06/13)
	• Trial conduct according to ICH-GCP-guidelines and the Declaration of Helsinki

**Treatment plan**	• First arm: ibuprofen 3 × 400 mg/3 days
	• Second arm: ciprofloxacin 2 × 250 mg, 1 × placebo/3 days

**Funding**	• German Federal Ministry of Research and Technology (BMBF)

## Methods

Feasibility was to be studied first since the German health care system lacks a central database of physicians or medical patient data that could be used for identification of participants or extraction of data. Correspondingly, we defined feasibility according to 1) successful practice recruitment including training and co-operation throughout the trial,

2) sufficient patient recruitment and follow up,

3) complete and accurate data collection and

4) appropriate protection of patient safety according to the ICH-GCP guidelines [9].

Additional aspects of feasibility - i.e. personal/time/financial resources - will also be considered.

The term "study team" will be applied to members of the University Departments' research teams consisting of two study nurses and two GP researchers (IG, JB). "Study nurse" represents a member of the study team, whereas "practice team" includes GPs and practice nurses.

The trial was supported by the Hannover Clinical Trial Centre (HCTC, a local co-ordination centre for clinical trials), which was involved in the preparation of the study protocol, materials, monitoring and data management.

### Practice recruitment, training, support

To achieve the target number of 30 participating practices, a convenience sample of 169 GPs in and around Hannover and Goettingen was invited by personal letters. Most of these practices had either previously been involved in research projects initiated by one of the two academic departments of general practice, or were members of their academic teaching networks. An academic GP researcher and a study nurse visited interested practices (pre-study-visit) to establish confidence and facilitate further co-operation. GPs and their practice teams were informed about the trial and its special requirements, such as GCP-guidelines, data collection, monitoring etc. An incentive of 100 € per completely documented patient was offered. The six-month patient recruitment period started with an initiation visit, performed by a staff member of HCTC in co-operation with the study team (GP researcher and study nurse). This visit provided practices with detailed instructions regarding study procedures and handouts of all trial related documents, tests and blinded trial medication. In particular, documentation within the case report form (CRF), reporting source data and adverse events and the procedure for deblinding in case of emergency were explained. Additionally, practice teams were urged to call the study team when they had any questions or problems concerning the RCT. During the recruitment period, regular visits to the practices by both an independent HCTC monitor and one of the research team's study nurses assessed the complete documentation with source data for each included patient. In addition, these visits were used to recapitulate trial workflow and documentation requirements. Paper CRFs were used instead of electronic CRFs as electronic documentation equipment within German practices is variable [10].

### Patient recruitment and follow up (see figure 1)

**Figure 1:**
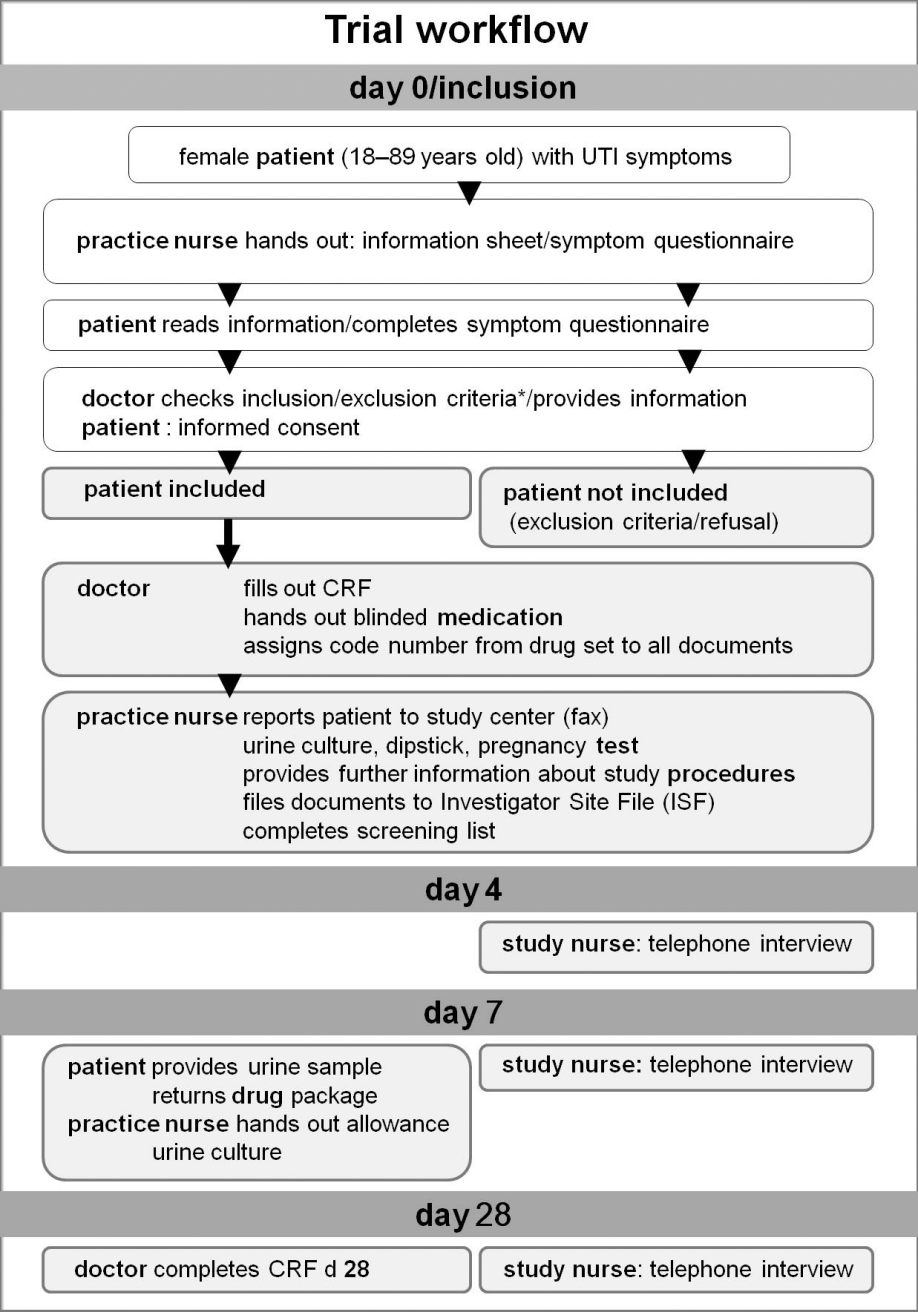
**Trial workflow**. Screening and enrolment procedures at inclusion Inclusion criteria: women with typical symptoms of UTI (dysuria and/or frequency). Exclusion criteria: any signs indicating a complicated UTI (i.e. fever, back pain), any conditions that may lead to complicated infections (i.e. pregnancy, diabetes, renal diseases, urinary tract abnormalities or past urinary surgery, urine catheterization, immunosuppressive therapy, other serious diseases, cancer), current use of antibiotics or non-steroidal anti-inflammatory drugs; history of gastrointestinal ulcers; epilepsy, allergies or other contraindications for trial drugs; inability to understand the trial information or to give informed consent.

The aim was to include at least 100 patients in the trial. Participating practices were requested to screen and document all adult female patients presenting with typical UTI-symptoms during a six month period. As German UTI patients usually consult their GP without a prior appointment or on very short notice, crucial study procedures as screening, enrolment and treatment had to be managed by practice teams without on-site support from the study team. Thus, every effort was made to prepare trial procedures compatible with practice routines as far as possible. A trial workflow and a checklist were provided to support the practice teams during the screening and enrolment procedures.

According to the workflow instructions, as practice nurses represented the first contact they were to inform patients about the study. Nevertheless, practices were allowed to modify this if it did not fit with their specific working routines. Practice teams were instructed to hand out trial information and symptom questionnaires to all eligible patients, including those who chose not to participate, so that we could assess the denominator for recruitment. Follow-up was to be conducted by the study nurses who performed structured telephone interviews on day 4, 7 and 28.

### Correct/complete data

CRF documentation at inclusion involved information on in- and exclusion criteria, a patient's history, basic clinical information including diagnoses and medication as well as pregnancy test and dipstick results. Completed symptom questionnaires and informed consent sheets had to be obtained and filed. During the follow-up period, urine culture results and information about potential adverse events (AEs) were to be documented in the CRF. In addition, all further patient-related information such as clinical findings or observations necessary for the evaluation of the trial (source data) had to be documented in patients' records. The practice team was informed of the importance of data quality, and notified of data review during the subsequent monitoring visits. Both the study protocol and patient information materials were developed with the support of HCTC to ensure compliance with regulatory requirements.

### Safety aspects

During trial participation all adverse events (AEs) were recorded, including conditions or symptoms where association with study medication seemed highly unlikely. Should AEs lead to a consultation, this had to be documented by the GPs; otherwise they were documented by the study nurses during follow-up telephone interviews. Approval by ethics committees (University of Goettingen and Hannover Medical School) and the German Institute for Pharmaceuticals and Medicine products (BfArM,) was obtained before starting the trial.

## Results

### Recruitment and cooperation with practices

Of all 169 invited GPs, 35 expressed an interest in the study and 29 (23 male, 6 female) finally agreed to participate. Main reasons for non-participation were lack of time or personal resources as well as ethical reservations regarding UTI management without antibiotics. A total of 28 GPs completed the six months recruitment period. One GP withdrew for ethical reasons citing non-antibiotic UTI management before patients were enrolled. With one exception, the GPs had no experience with drug trials according to GCP.

Both the pre-study visits and initiation visits informed the practice team of study details and helped establish a working relationship with the study team. However, although GPs and practice staff showed an interest in the study in general, many of them seemed to be overwhelmed by the amount of information and regulations, as clinical trial procedures differ significantly from observational or educational intervention studies. In particular, GPs were astonished by the procedural complexity associated with both the documentation and reporting of AEs and of source data (as required by German law).

During a total of 52 monitoring visits (1-3 per practice, duration 2-4 h), lists of errors and missing data entries were presented to the GP, which had to be corrected. Some GPs seemed to be glad that errors had been detected and corrected; others resented "being controlled". Since an independent monitor had to be involved trial costs for initiation and monitoring were on average 600 € per monitoring visit. Concerning study procedures, practice teams commented repeatedly that they felt well supported by the workflow instructions and that the checklist helped them to remember study procedures during their everyday routine.

In conclusion, recruitment of and co-operation with practices for this drug trial were successful. However, both GPs and academic study teams had to invest considerable resources in terms of personnel and time for training and supervision.

### Patient recruitment

Patient recruitment and follow-up: A total of 195 UTI patients were documented as having been screened, 65 had exclusion criteria and 50 refused to participate. Excluding one screening failure, 79 patients were finally enrolled - less than our target sample size of 100 patients. Recruitment differed strongly between practices (range 0-12); 3/29 practices had no study participants, 9/29 recruited only one patient whereas only two practices enrolled 10 or more patients. The average recruitment rate was 2.8 patients per practice. None of the patients cancelled study participation during either treatment or follow up. GPs who did not recruit patients within a period of eight weeks were contacted and offered further training. The most frequently reported reasons for poor recruitment given during phone calls were: no eligible cases, busy times in the practice or practice vacation. Many GPs had overestimated their potential for patient recruitment and as anticipated, practice nurses became the key figure in screening und recruitment of patients; they were usually the first contact person. However, the additional time required for screening and enrolment within a normal consultation routine proved a considerable obstacle to patient recruitment - most practice nurses and general practitioners admitted that they had forgotten trial screening in busy times. Thus, it is not possible to reliably state the number of eligible patients. Further feasibility issues in terms of trial requirements and related problems are reported in table 2.

**Table 2:** Trial requirements and related problems.

Investigator duties (GCP)	Provided by the study team	Problems encountered	Possible causes
Knowledge of ICH-GCP guidelines and regulatory requirements	• *Information (ICH-GCP guidelines, German drug law etc.)* • *Training units within initiation/monitoring visits* • *Further training units on demand*	• *Written material was not read*	• *Too much information provided in initiation visits*

Providing resources (time, staff) Clarify responsibilities	• *Workflow support* • *Additional support on request (by phone call, additional visits)*	• *Lack of personal resources (during busy practice times)*	• *Under-estimation of additional trial workload* • *Recruitment of incident cases*

Sufficient patient recruitment (screening, enrolment and documentation of all UTI patients)	• *Involvement of practice nurse* • *Support by instructions, workflow*	• *Low recruitment in some practices* • *Incomplete documentation for non-participants*	• *Time constraints* • *Time gap between initiation visit and first inclusion* • *Number of refusals/patients with exclusion criteria under-estimated*

Comply with regulatory requirements with respect to informed consent	• *Extensive but GCP-conforming information sheets*	• *Patients felt overwhelmed with information* • *Patients did not read information completely before signing* • *Time-consuming procedure*	• *Information sheet too extensive*

Ensure complete and correct documentation (CRF, source data)	• *Support by instructions, workflow* • *Data sheets for relevant source data*	• *Incomplete CRFs* • *Problems with report of AEs*	• *Extensive documentation not compatible with existing documentation habits*

Abbreviations: CRF = case report form, ISF = investigator site file, AE = adverse event

### Data collection

On day 0, 72 of 79 symptom questionnaires had been completed properly and were available for analysis. In seven cases, practice teams had forgotten to collect symptom questionnaires. In many practices, data had to be completed with support from the study nurses. CRF documentation during follow up (e.g. AEs and urine culture laboratory results) in particular was sometimes simply forgotten and had to be collated later.

As for symptom course, follow-up-data from telephone interviews were available in 77 (day 4), 76 (day 7), 76 (day 28) of 79 cases, respectively. The omissions arose as some patients could not be contacted by phone. Source data documentation in patients' records was often inadequate but this could be resolved by providing an extra source data sheet to capture the extra information.

### Safety

A total of 58 AEs were retrieved during the telephone interviews, which were mainly described as slight discomfort and did not necessarily lead to a further consultation with the GP. The most commonly reported symptoms were nausea, vomiting and diarrhoea (15/58), followed by upper respiratory complaints (11/58). GPs documented 23 AEs in the CRFs, which represented all the adverse reactions that led to a GP consultation during the 28 days of the study participation. Altogether, the majority of the documented symptoms seemed to be coincidental rather than directly related to the study treatment. In two cases, serious AEs resulting in hospital admissions were reported - one patient admitted herself with abdominal pain during the trial participation (nothing serious was found, and she was discharged after two days); another was admitted with sudden hearing loss. No sudden unexpected serious adverse reactions (SUSARs) were observed during the trial.

## Discussion

This double-blind randomised controlled drug trial in German general practices proved feasible in terms of both practice and patient recruitment, as well as with respect to data quality and patient safety. However, due to a lack of research training or familiarity with established procedures (i.e. in documentation) and little previous experience of drug trials, the participating practices required significant input from the academic study team. Their willingness to invest extra time and effort on behalf of the practices was essential to the success of this study.

Barriers to performing an RCT in general practices have been described before: Time constraints, elaborated study procedures during routine consultations or a GP's lack of interest in the study theme can all hinder patient recruitment [11-14]. In particular, studies requiring incident cases are known to be less successful [15]. This is in line with our experience: patient recruitment during regular consultation hours managed by the GP and practice staff in the absence of on-site support by study nurses represents a major challenge both for GPs and the study team - and is likely to cause problems.

We additionally found that briefing GPs on GCP basics within a short time (initiation visit) kept not only the threshold for participation relatively low but also created an additional problem as the information was not sufficiently retained. The study team provided phone support to ensure correct AE reporting. However, during the trial regulations in Germany were tightened, and now all investigators in clinical drug trials must attend a certified GCP-training course. This may, in itself, become a real obstacle as courses are time-consuming and costly, and currently tailored more to the needs of hospital-based investigators than GPs. It remains unclear whether GPs can be expected to pay for these out of their own pocket. In our experience, the academic research team has an essential role in detailing study requirements to the practice teams. As shown in table 3, knowledge of practices' working conditions and close contact to the GP and his practice team are important for a successful trial.

**Table 3:** Further implications for practice

Implications for practice
• Support recruitment: through close contact to GPs, reminders, adequate reimbursement • Keep trial procedures as simple and flexible as possible: checklists and workflow instructions should be adapted to local practice habits • Consider key roles: involve practice nurse in incentives and training to optimise workflow • Remember that time constraints are a major reason for insufficient recruitment: make this a subject of discussion with the GPs and try to find solutions • Remember that the academic research team have an essential role in detailing study requirements to the practice team, as well as practice habits to external monitoring organisations • Keep patient information as simple as possible • Consider risk-adapted monitoring • Prepare measures to optimise source data documentation (source data sheets)

Quality management to ensure that correct procedures and documentation are used through the trial requires monitoring visits. These represent a considerable burden for participating GPs and high costs for the trial. Options for risk-adapted monitoring (ADAMON) as described recently by Brosteanu et al. [16] should be considered for non-commercial trials comparing the effectiveness of established treatments and licensed drugs.

Regulatory requirements: Though practice related procedures could be optimised by thorough preparation, many regulatory demands seemed to be over the top and have built up barriers rather than improved data quality. Considering for example that the trial compared two licensed, well-established treatments taken for 3 days, one of which is available over the counter, demands on AE reporting were hard to justify to GPs. As a result, many GPs did not implement procedures such as prompt and detailed documentation reporting any patient discomfort. This was also often the case for source data documentation as it is unusual for a GP to comment on conditions their patients do not have (though this is mandatory in trials if the condition is an exclusion criterion). GPs also reported that patients with uncomplicated UTI found the legally required GCP paperwork to give their consent to participate in the trial complicated and that most did not seem to read this carefully.

The problems related with extensive clinical trial regulations have been discussed by many researchers in conferences and working groups in recent years [17,18]. It seems undisputed that regulatory demands, while aiming to insure maximum patient safety, also create a maximum of documentation and bureaucracy. In case of trials comparing licensed, commonly used drugs generally considered as safe, this may be excessive, and hamper trials rather than improve their quality [19-21]. As a consequence, researchers demand a risk-adapted approach of GCP-regulations and sensible guidelines to simplify the conduct of trials when risk can be considered low [22,23]. Non-commercial comparative effectiveness trials are particularly important both as a contrast to commercial research interests and as a source of urgently needed evidence for safe, effective and cost-effective treatment options in primary care [24-26].

Strengths and Limitations: The main strength of this paper refers to its "novice" setting in which feasibility, barriers and facilitators of a clinical trial are reported by combining study data, more narrative process data and experience. On the other hand, this may also constitute a limitation. This RCT was conducted as a pilot trial with a relatively small sample size. Our experience cannot be considered representative and should be interpreted with respect to this context and setting.

## Conclusions

To enable drug trials in German general practice, it will be necessary to adapt the setup of clinical research infrastructure to the needs of GPs and their practice staff. To support non commercial clinical comparative effectiveness trials in primary health care, adaption of clinical trial regulations to the risk engendered by the trial seems necessary. For low risk trials using licensed drugs, administrative procedures could probably be reduced without compromising patient safety.

## Abbreviations

ADAMON: adapted monitoring concept; AE: adverse event; BMBF: Bundesministerium für Bildung und Forschung (German Federal Ministry of Research and Technology); BfArM: Bundesinstitut für Arzneimittel und Medizinprodukte (German Institute for Pharmaceuticals and Medicine Products); CRF: case report form; GCP: Good Clinical Practice; GP: General Practitioner; HCTC: Hannover Clinical Trial Centre; HWI: Harnwegsinfekt (urinary tract infection); ICH: International Conference on Harmonisation; ISF: investigator site file; RCT: randomized clinical trial; SUSAR: suspected unexpected severe adverse reaction; UTI: urinary tract infection; UK: United Kingdom.

## Competing interests

The authors declare that they have no competing interests.

## Authors' contributions

EHP and MMK had the original idea for the study. IG and JB recruited and supervised the practices and managed the trial on a day-to-day basis. Data analysis was performed by KW. All authors read and approved the final manuscript.
